# Learners’ Perception of the Applicability of Pre-clinical Subjects to Clinical Training and Experience in Obafemi Awolowo University, Ile-Ife

**DOI:** 10.15694/mep.2020.000138.1

**Published:** 2020-06-29

**Authors:** Beatrice Emma-Okon, Ganiat Omoniyi-Esan, Josephine Eziyi, Olufemi Ogundipe

**Affiliations:** 1 Obafemi Awolowo University

**Keywords:** Perception, Applicability, OAU Medical School, Anatomy, Biochemistry, Physiology, Curriculum

## Abstract

This article was migrated. The article was marked as recommended.

**Introduction:** This study investigated how Final Year Students and Newly qualified Doctors of Obafemi Awolowo University (OAU) Medical School perceive the applicability of the three pre-clinical subjects (Anatomy, Biochemistry and Physiology) to clinical studies and medical practice with the aim of ascertaining whether or not the students are able to establish a real connect between knowledge acquired in the pre-clinical years and their experience during clinical exposure.

**Methods:** A cross sectional study involving 106 final year medical students and 81 interns was conducted using convenience sampling method. Data was collected using a structured self-administered questionnaire consisting of demographic characteristics and 10 items regarding perception of the relevance of basic medical courses to clinical training experience. Responses were rated using a 5 point Likert scale, which ranged from “strongly disagree” to “strongly agree”. Data analysis was carried out using descriptive and inferential statistics and values expressed as mean of scores.

**Results:** Overall mean scores for anatomy, biochemistry and physiology were 26.4 ± 0.32, 25.7 ± 0.29 and 28.1 ± 0.31 respectively. Thus, physiology was rated as having the highest applicability with the highest score in 4 out of 10 items. Biochemistry had the lowest score for the item “I remember most of the content of the course even now”. Newly qualified Doctors were found to have a more positive perception of anatomy and biochemistry than final year medical students. All Subjects were scored high (average of 3-0/4.0) on items having to do with introduction of curriculum integration and clinical studies to teaching.

**Conclusion:** Findings from the study suggest that the responses of the subjects were based more on their ability to understand and recall content, rather than how relevant the subjects are to their clinical experience. The study concludes that there is an urgent need to embrace integration of the curriculum and introduce more learner-centered teaching methods.

## Introduction

Sound knowledge and understanding of the basic medical sciences provides a rational basis that can be adapted for the practice and advancement of medicine. In this light, the pre-clinical subjects (Anatomy, Physiology and Biochemistry) are presumed to be essential for the understanding and practice of the clinical sciences, hence these subjects are taught to medical students before they proceed to the clinical years. In Obafemi Awolowo University, these subjects are the only courses taken in the second and third years of Medical School training. It is important to study the perception of learners as far as applicability and relevance of these subjects to clinical studies are concerned in view of the fact that students’ achievements and future practice are affected by their attitude towards previous knowledge ( Bean and Eaton, 2002; Lizzio, Wilson and Simons, 2001). In addition, the courses are taught in their most basic forms with a lot of emphasis on the theoretical aspects and the interval between learning the theory and its usage in the clinic could be a potential problem in the recall of the knowledge and ability to apply it to clinical settings ( Larsen, Butler and Roediger, 2013). It is imperative to ensure that students are able to establish a real connect between knowledge acquired in the pre-clinical years and their clinical exposure and eventual practice. Recently in Nigeria, the National Universities Commission (NUC) proposed an integrated curriculum for all colleges of Health Sciences in the country, in a bid to enrich learning experience and facilitate development of knowledge that is relevant and meaningful to clinical practice ( Federal Ministry of Health, 2012) but this is yet to be implemented in Ife Medical School.

This study is aimed at determining how medical students rate their ability to retain knowledge of the basic medical subjects and its applicability to clinical training and attempts to answer the question “Is curriculum integration necessary for our students”?

## Methods

A cross sectional study was conducted in the College of Health Sciences, Obafemi Awolowo University, Ile Ife among 106 final year medical and dental students and 81 interns regarding their perceptions of the relevance of pre-clinical basic sciences in clinical training experience. Convenience sampling method was used and verbal consent was obtained prior to their participation in the study.

Data was collected using a structured self-administered questionnaire. The first part included students’ demographic characteristics such as age, gender and academic year while the second part consisted of 10 items regarding the respondents’ perception of the relevance of basic medical courses in their clinical training experience. The items listed on the questionnaire were adapted from a study carried out in Bangladesh ( Atukolara and Atapattu, 2014). All responses were rated using the 5 point Likert scale, which ranged from “strongly disagree” to “strongly agree”. The answer “strongly disagree” was scored as 0 and “strongly agree” as 4. Negative statements (items 4 & 5) were scored in reverse order. Data analyses was carried out using descriptive and inferential statistics. Values were expressed as mean of scores. T-test was used for comparison of continuous variables. Data were presented as mean ± SD. Statistical significance was determined at *P* < .05. All analyses were performed with SPSS version 20.

## Results/Analysis

A total of 187 students and newly qualified Doctors were included in this study. Age range was between 22 and 39 with a mean of 25.2 ± 2.6. Most of the respondents 137(73.3%) were less than 25 years, 35(18.7%) were between 25 and 29 years while the remaining 15(8.0%) were 30 years or older. Most of the participants were male (n =137, 73.32%) and were in final year of study (n =106, 56.7%). Demographic characteristics are presented in Table 1.

**Figure 1.**
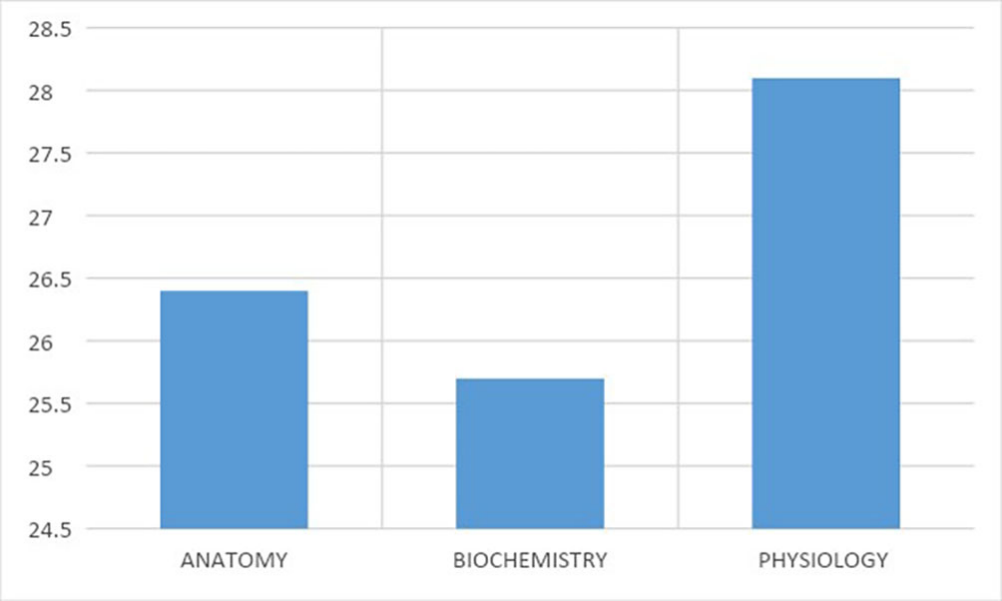
Overall Scores for perception of Applicability of Pre-Clinical Subjects

The overall mean scores for perception of applicability for each preclinical course are reported in Table 2. The overall mean scores for anatomy, biochemistry and physiology were 26.4 ± 0.32, 25.7 ± 0.29 and 28.1 ± 0.31 respectively ( Figure 1). Thus physiology was rated as having the highest applicability overall score with the highest score in 4 out of 10 items (items 2, 3, 4 and 8). Table 3 indicates comparison of responses between final year students and newly qualified Doctors. Only items with significant differences in response between the two groups are shown. While newly qualified Doctors have higher scores (better perception) of applicability of Anatomy and Biochemistry, the reverse is the case for Physiology.

No significant differences were found when responses were grouped according to gender.

**Table 1.** Demographic Characteristics of Respondents

Characteristics	All participants (n= 187) Frequency (%)	Clinical 3 students (n = 106) Frequency (%)	NQD (n = 81) Frequency (%)
**Sex**			
Male	137 (73.3)	78 (73.6)	59 (72.8)
Female	50 (26.7)	28 (26.4)	22 (27.2)
**Age group (in years)**			
>25	137 (73.3)	84 (79.3)	53 (65.4)
25 - 29	35 (18.7)	12 (11.3)	23 (28.4)
30 ^+^	15 (8.0)	10 (9.4)	5 (6.2)
Mean age (SD) years	25.2 (2.6)	24.9 (2.8)	25.5 (2.3)

**Table 2.** Scoring of Perception of Applicability of Pre-Clinical Subjects

	Applicability	Subjects		
		Anatomy	Biochemistry	Physiology
		Mean (SE)	Mean (SE)	Mean (SE)
1	Educational activities provided prepared me well for the required clinical learning experience	2.0 (0.09)	2.9 (0.07)	2.1 (0.08)
2	The course content was useful during my clinical training	2.5 (0.07)	2.5 (0.07)	3.1 (0.06)
3	I was /have been able to apply most of the knowledge acquired to my practical experience in clinical years	2.4 (0.08)	2.3 (0.08)	2.9 (0.07)
4	The presentation did not adequately bring out the clinical relevance	1.8 (0.08)	1.9 (0.08)	2.7 (0.07)
5	The curriculum is overloaded with a lot of rarely clinically applicable content	2.6 (0.09)	2.7 (0.08)	1.9 (0.09)
6	There is a need for curriculum integration of the Basic Medical Sciences with Clinical Sciences	3.4 (0.06)	3.4 (0.06)	3.3 (0.06)
7	I had to frequently revisit the subjects during my Clinical training	3.0 (0.07)	2.6 (0.07)	2.7 (0.07)
8	I remember the content of the Course even now	1.9 (0.08)	1.7 (0.08)	2.5 (0.09)
9	Some clinical case studies should be introduced to the teaching	3.5 (0.05)	3.4 (0.05)	3.3 (0.06)
10	Some clinical practice should be introduced to the teaching	3.4 (0.06)	3.4 (0.6)	3.3 (0.06)
	**Gross score**	**26.4(0.32)**	**25.7(0.29)**	**28.1(0.31)**

**Table 3.** Significant differences in perception of applicability of pre-clinical subjects among final year students and newly qualified Doctors

Applicability	Subjects											
	Anatomy				Biochemistry				Physiology			
	CLI3	NQD	t-test	p-val	CLI3	NQD	t-test	p-val	CLI3	NQD	t-test	p-val
Educational activities provided prepared me well for the required clinical learning experience	1.70	2.31	3.51	<0.01	1.96	2.21	1.57	0.11	3.01	2.68	2.34	0.020
The course content was useful during my clinical training	2.51	2.81	2.12	0.035	2.44	2.62	1.17	0.24	3.25	2.98	2.35	0.020
I was /have been able to apply most of the knowledge acquired to my practical experience in clinical years	2.31	2.59	1.85	0.066	2.23	2.36	0.86	0.386	3.10	2.67	3.03	0.003
I remember the content of the Course even now	1.70	2.05	2.25	0.025	1.63	1.84	1.30	0.192	2.58	2.37	1.38	0.168
Some clinical case studies should be introduced to the teaching	3.54	3.41	1.19	0.236	3.49	3.31	1.77	0.078	3.44	3.17	2.38	0.018
Some clinical practice should be introduced to the teaching	3.55	3.25	2.57	0.011	3.45	3.21	2.22	0.028	3.43	3.07	3.03	0.003

## Discussion

This study was aimed at determining how final year students and newly qualified doctors of Obafemi Awolowo University College of Health Sciences perceive the applicability and relevance of Pre-clinical subjects (Anatomy, Biochemistry and Physiology) to clinical studies. Learners’ perception and views of the importance, relevance and applicability of course taught is important in making necessary changes in order to enhance learning experience. The choice of newly qualified doctors and final year students for the present study is based on the fact that these two groups have the benefit of having recently gone through both the pre-clinical and clinical years and so are in a good position to recall and give account of how they have been able to connect both experiences. Similar previous studies have also focused on this group in making attempts to investigate perception of pre-clinical teaching, learning environment and relevance to clinical settings ( Atukolara and Atapattu, 2014; Gupta *et al.*, 2014; Fitzgerald *et al.*, 2008; Shankar *et al.*, 2007).

While it is well accepted that the three pre-clinical subjects are important in preparing medical students for clinical studies and practice, it is important to ascertain how learners rate these subjects in terms of relevance and applicability. In this study, Physiology obtained the highest score (28.1 out of 40) when the mean scores for the 10 items were added. Items for which Physiology was rated highest include “course content was useful during my clinical training” (3.1 out of 4.0 compared to 2.5 for anatomy and Biochemistry), “I have been able to apply most of the knowledge acquired to my practical experience in clinical years” (2.9 out of 4.0 compared to 2.4 for anatomy and 2.3 for biochemistry) and “I remember the content of the course even now” (2.5 out of 4.0 compared to 1.9 for anatomy and 1.7 for biochemistry). The respondents also believed that physiology curriculum brought out the clinical relevance better than anatomy or biochemistry. In a study carried out in Saudi Arabia among Medical students in their clinical years, Anatomy and Pathology were identified as the courses most overloaded with content while half of the students felt that they retained knowledge most in Physiology and only 19% felt they retained knowledge most in biochemistry. Physiology was also perceived as the subject with the highest applicability to clinical practice ( Alam, 2011). Other studies in which Physiology was perceived as being the most clinically relevant subject include those of Atokolara and Atapattu (2014) and Bryant, Sen and Sood (2014).The fact that Physiology was perceived to be most relevant as well as most retained confirms the belief that perceived relevance of a subject fosters retention of knowledge ( Spencer and Jordan, 1999; Malau-Aduli *et al*., 2013).

Interestingly, Anatomy was rated highest for the item “I had to frequently revisit this subject during my clinical training” and Biochemistry for the item “Educational activities prepared me well for the required clinical learning experience”. This suggests that their perception of the subjects was based more on their ability to understand and recall the content, rather than how relevant it is to their clinical experience i.e their perception of “most relevant” could just be on the basis of how easy it was for them to grasp the subject in terms of simplicity of language of expression. For example, Biochemistry is a rather theoretical subject in which the student has to learn many pathways, reactions and chemical processes and this is why medical students tend to shy away from it.

In this study, Biochemistry had a score of 1.7 out of 4.0 for the item “I remember the content of the course even now”. This shows that recall of knowledge for this subject is poor. However, things can be made easier with more practical sessions, introduction of clinical correlations, removal of excessive details, introduction of self-learning methods such as group presentations, assignments and by embracing the integrated curriculum. In fact, some adjustments and changes to teaching method may translate the perception of learners about this subject. Furthermore, it should also be noted that all three subjects were scored high (average of 3-0/4.0) on items having to do with introduction of curriculum integration and clinical studies to teaching. This underscores an urgent need to embrace integration of the medical curriculum as is the practice in some Medical schools. Integration of curriculum has been defined as education that is organized in such a way that it cuts across subject matter lines, bringing together various aspects of the curriculum into meaningful association to focus on broad areas of study ( Shoemaker, 1989). The advantages of such integration include an integrated and harmonized view of topics which enhance understanding, introduction of more clinical content and correlations, a shift from faculty-centered to student-centered learning with an increase in small group discussions, tutorials and students’ use of the library for self-directed learning and reduction of over-dependence on lectures for the transfer of knowledge.

From Table 3, it can be inferred that newly qualified Doctors have a more positive perception of anatomy and biochemistry than final year medical students as far as applicability is concerned. This further confirms the fact that all three subjects are in reality relevant to clinical practice and because the young Doctors are already involved in Medical practice, they are better able to appreciate the importance of all the pre-clinical subjects ( Quintero *et al.*, 2016; Gole, Meshram and Hattangadi, 2015).

## Conclusion

Although learners in OAU Medical school believe that all three pre-clinical subjects are relevant to clinical studies, Physiology is perceived as the most relevant and Biochemistry as the least relevant. It is also obvious that the much advocated integration of learning is long overdue in OAU Medical school, although learners must realize that this will by no means be a short cut to achieving success in Medical school. The School also has to ensure that modalities are properly put in place for this new learning system to be implemented. This will include staff training, provision of e-learning facilities and recruitment of more staff to facilitate teaching in smaller groups.

## Take Home Messages

• A good working knowledge of Anatomy, Biochemistry and Physiology is required for clinical studies and medical practice
• Learners in OAU Medical School believe that Physiology is the more relevant to clinical settings than Anatomy and Biochemistry
• Integration of the medical curriculum and introduction of more learner-centered methods of teaching will help students to establish a connect between these subjects and clinical training and experience

## Notes On Contributors

**B.O Emma-Okon** is a Senior Lecturer and the Acting Head of the Department of Medical Biochemistry. She has taught Biochemistry in the College of Health Sciences for 16 years. Her Students include 2 ^nd^ and 3 ^rd^ year Medical, Dental, Physiotherapy and Nursing Students. She is also an external examiner to some Medical Schools. ORCID: https://orcid.org/0000-0001-8739-7175

**G.O Omoniyi-Esan** is a professor of Morbid Anatomy in the College of Health Sciences and a Consultant Pathologist at the OAU Teaching Hospitals. She holds a Masters degree in Medical Education and is a fellow of the Sub-Saharan Africa Faimer Regional Institute (SAFRI).

**O.K Ogundipe** is a Senior Lecturer in Obafemi Awolowo University and Honorary Consultant in oral and Maxillofacial Surgery at the OAU Teaching Hospitals Complex. He has taught undergraduate Dental Students at pre-clinical and clinical levels for 4 years. ORCID: https://orcid.org/0000-0003-4928-6970

**J.A.E Eziyi** is an Associate Professor and a consultant ENT-HNS Surgeon. She has a fellowship in Medical Education with special interest in Students’ assessment (blueprinting).

## Declarations

The author has declared that there are no conflicts of interest.

## Ethics Statement

The Management of the College of Health Sciences, Obafemi Awolowo University has granted permission for the study findings to be published.

## External Funding

This article has not had any External Funding

## References

[ref1] Alam A. (2011) How do Medical Students in their clinical years perceive basic sciences courses at King Saud University? Ann Saudi Med. 31(1), pp.58–61. https://dx.doi.org/10.4103%2F0256-4947.75780 21245601 10.4103/0256-4947.75780 PMC3101727

[ref2] Atokorala K. R. and Atapattu P. (2014) Pre-clinical Basic Sciences Teaching curriculum of a Medical School in a developing country - Are we doing it right? J Bangladesh Soc Physiologist. 9(2), pp.98–104. 10.3329/jbsp.v9i2.22806

[ref3] Bean J. and Eaton S. B. (2002) The Psychology underlying successful retention practices. J College Student Retention. 3(1) pp.73–89. https://doi.org/10.2190%2F6R55-4B30-28XG-L8U0

[ref4] Bryant J. Sen R. and Sood S. K. (2014) Undergraduate Medical Students’ perceptions and opinions towards the subject of Physiology. International Journal of Biomedical and Advance Research. 5(12), pp.605–608.

[ref5] Federal Ministry of Health, Nigeria . (2012) Health Systems 20/20 project, Nigeria Undergraduate Medical and Dental Curriculum Template. Available at: https://www.hfgproject.org/wp-content/uploads/2015/02/Nigeria-Undergraduate-Medical-and-Dental-Curriculum-Template.pdf(Accessed: 06 April 2020).

[ref6] Fitzgerald J. E. F. White M. J. Tang S. W. Maxwell-Armstrong C. A. (2008) Are We Teaching Sufficient Anatomy at Medical School?: The Opinions of Newly Qualified Doctors. Clinical Anatomy. 21, pp.718–724. 10.1002/ca.20662 18773486

[ref7] Gole R. A. Meshram P. and Hattangdi S. (2015) Changes in perception about Anatomy subject after 1st year of medical course. India J Basic Appl Med Res. 4(4), pp.453–457.

[ref8] Gupta S. Gupta A. K. Verma M. Kaur H. (2014) The attitudes and perception of medical students towards basic science subjects during their clinical years: A cross-sectional survey. Int J Appl Basic Med Res. 1, pp.16–19. 10.4103/2229-516X.125675 PMC3931207 24600572

[ref9] Larsen D. P. Butler A. C. and Roediger H. L. (2013) Comparative effects of test-enhanced learning and self-explanation on long term retention. Medical Education. 47, pp.674–682. 10.1111/medu.12141 23746156

[ref10] Lizzio A. Wilson K. and Simons R. (2002) University Students’ perceptions of the learning environment and academic outcomes: Implications for Theory and Practice. Studies in Higher Education. 27(1), pp.27–52. 10.1080/03075070120099359

[ref11] Malau A. Bunmi S. Lee A. Catchpole M. (2013) Linking basic science knowledge retention and perceived clinical relevance in a vertically integrated curriculum.In: Abstracts from AMEE 2013. 2D/1. pp. 9. From: AMEE 2013: An International Association for Medical Education, 24-28 August 2013. Prague Czech Republic. Available at: https://amee.org/getattachment/Conferences/AMEE-Past-Conferences/AMEE-Conference-2013/AMEE-2013-ABSTRACT-BOOK-updated-190813.pdf (Accessed: 06 April 2020).

[ref12] Quintero G. A. Vergel J. Arredondo M. Ariza M. (2016). Integrated Medical Curriculum: Advantages and Disadvantages. J. Med Edu and Curr Devt. 16(3), pp.133–137. https://doi.org/10.4137%2FJMECD.S18920 10.4137/JMECD.S18920 PMC5736212 29349303

[ref13] Shanka P. R. Dubey A. K. Subish P. and Upadhay D. K.,(2007) Medical Students’ attitudes towards and perception of the basic sciences in a medical college in western Nepal. Med Sci Educ. 17, pp.85–l92. Available at: https://www.researchgate.net/publication/267327979 (Accessed: 06 April 2020).

[ref14] Shoemaker B. J. E.,(1989) Integrative Education: A curriculum for the twenty-first century. OSSG Bulletin. 33(2),n2. Available at: https://eric.ed.gov/?id=ED311602 (Accessed: 06 April 2020).

[ref15] Spencer J. A. and Jordan R. K. (1999) Learner centered approaches in medical education. BMJ. 318(7193), pp.1280–1283. 10.1136/bmj.318.7193.1280 10231266 PMC1115656

