# Supplementary material for: Learners’ Perception of the Applicability of Pre-clinical Subjects to Clinical Training and Experience in Obafemi Awolowo University, Ile-Ife
Source: MedEdPublish (2016). 2020 Jun 29;9:138. [Version 1] doi: 10.15694/mep.2020.000138.1 (PMC10702685; doi:10.15694/mep.2020.000138.1)
Supplement: Peer review history

# Reviewer report: Reviewer response for version 1

**DOI:** 10.21956/mep.20035.r30657

**Published:** 2020-07-06

**Authors:** Ben Canny

**Affiliations:** 1 Monash University

This review has been migrated. The reviewer awarded 3 stars out of 5 This interesting manuscript reports final year students’ and newly qualified doctors’ perceptions of the applicability of “pre-clinical” subjects to clinical training and experience. A significant strength of the manuscript is the large numbers of participants (106 students and 81 interns). I have concerns, however, about how the data are handled, and the statistical analysis was undertaken and therefore the conclusions drawn from the data collected.The Likert-scale results from 10 questions were combined to give an “overall (gross)” score for the subject (Table 2 and Figure 1). It is concluded that Physiology is perceived to be more applicable than Anatomy or Biochemistry, though no statistical comparison is reported. Combining the scores implies that each question is measuring an outcome that is directly comparable, and there is a high degree of comparability in the scale of the score (ie the difference between two points on the Likert scale means the same for separate questions). It is unlikely that these criteria are satisfied. For instance, Q1, Q2, Q4, Q9 and Q10 relate to pedagogical issues, where Q2 and Q3 relate to clinical applicability. Other differences between questions probably also exist.Table 3 reports statistical differences between the perceptions of final year students and newly qualified doctors. It appears that multiple t-tests were conducted (possibly at least 18, but 30 may have been undertaken) without any reported corrections for Type 1 statistical errors. It is worth noting that with 18 t-tests, and a fixed alpha value of 0.05, there is a greater than 60% chance of at least one test being significant.These statistical issues could be overcome by employing a repeated errors analysis of variance, where data are analysed for each question, with the within-factors variables being the subjects and the questions, with a between-factors variable of final year students vs newly qualified doctors. The authors have access to SPSS, which is capable of this analysis, and many statisticians would be able to advise regarding the analysis and post hoc testing.These comments are made to help in possibly strengthening the conclusions of this interesting manuscript. For instance, the authors may be able to report, with greater confidence, whether the responses to Q6, Q9 and Q10 are, indeed, more positive than other questions, strengthening the argument for integration.

Reviewer Expertise:

NA

No decision status is available

# Reviewer report: Reviewer response for version 1

**DOI:** 10.21956/mep.20035.r30655

**Published:** 2020-07-04

**Authors:** P Ravi Shankar

**Affiliations:** 1 American International Medical University

This review has been migrated. The reviewer awarded 4 stars out of 5 This is an interesting and well-presented study from Nigeria. The authors have studied the perceptions of final year students and newly graduated doctors toward the preclinical subjects of Anatomy, Biochemistry and Physiology. The article can benefit by a description of the existing medical program at the university and the proposed modifications to the curriculum. The authors rightly mention that the teaching-learning of Biochemistry can be modified with a greater emphasis on clinical relevance and application of the subject. The authors can also provide more information about the students admitted to the undergraduate medical program at the university and the recruitment process. Do students have to complete any mandatory rural service after graduation? How old is the University? Have there been previous modifications to the curriculum? The authors can provide more information about the ethical dimensions of their study. Which body provided ethical approval and why did they not obtain written consent from the participants? What percentage of the final year students and the newly graduated doctors participated? The article will be of interest to all medical educators and curriculum planners.

Reviewer Expertise:

NA

No decision status is available

# Reviewer report: Reviewer response for version 1

**DOI:** 10.21956/mep.20035.r30658

**Published:** 2020-07-02

**Authors:** Barbara Jennings

**Affiliations:** 1 Norwich Medical School

This review has been migrated. The reviewer awarded 3 stars out of 5 In this article, the authors study the perception of learners about the relevance of preclinical studies of the basic sciences physiology, anatomy and biochemistry. The context and motivation for the study is planned curricular change to integrate the undergraduate medical curriculum within an institution, in response to a national initiative. A cross sectional survey was completed for undergraduate final year students and recently qualified doctors. In their methods section the authors outlined the style of questions used in their structured self-administered questionnaire and explain that they were developed from a pre-validated tool. The questions themselves could be found by reviewing Table 2 in the results section. Perhaps, the methods section could have been more detailed and expanded to provide more information of participant recruitment and the research governance steps? With online publication it is straightforward to include information sheets and questionnaire documentation as supplementary files and appendices.The results led to some interesting observations about the difference in perception between final year students and those actually doing the job. I had some unresolved questions as I reviewed the methods and results sections. Could finals preparation have had any impact here and could study design have been improved by longitudinal follow up of the same participant cohort over two years. Were corrections made by the authors for multiple comparisons as part of their statistical analysis?Participants were most interested in long term knowledge gained from their earlier science classes and the authors stress a need for learner-centred approaches to life science education including integration with clinical practice.I was interested in how the different subjects were perceived by the participants (e.g. physiology was seen as useful to clinical practice and biochemistry knowledge was not well retained), anecdotally I notice related preferences and perceptions in undergraduate student cohorts in our integrated medical curriculum – student selected components for Physiology are usually over-subscribed while it is hard to recruit students to options in Biochemistry.I enjoyed reading this clearly written article and think it will be of interest to other teachers of life sciences in medical schools.

Reviewer Expertise:

NA

No decision status is available

# Reviewer report: Reviewer response for version 1

**DOI:** 10.21956/mep.20035.r30656

**Published:** 2020-06-30

**Authors:** Puja Dulloo

**Affiliations:** 1 Pramukhswami Medical College

This review has been migrated. The reviewer awarded 4 stars out of 5 The article is of my interest since I am a pre-clinical faculty and strongly believe that basic science is a foundation for the clinical science part of medical or other health professional education. If this foundation is week then top building of clinical cognition and skills will not be what is expected. Students might feel that their grades are enough to make them the best, but their logical and critical thinking approach would be missing at various clinical points, throughout their life. To be better physician they would be investing more time to better understand and interpret the clinical case, since they would be bushing around the foundation concept of the case.The study participants also agreed to the fact that knowledge of basic since is relevant in clinical course irrespective of difference in the mean score. This difference probable might be based on the type of patient dealt by them during the clinical rotational posting. Students entering medical programme should be shown the response of their senior students, as a reference, before they are taught pre-clinical subjects. Probably then they might take it more seriously.To add higher dimension, a qualitative study should have been done via semi-structured interview. I am sure authors would have identified newer stories from both the participant groups.I enjoyed reading this article.

Reviewer Expertise:

NA

No decision status is available
